# [Corrigendum] Zoledronic acid sensitizes breast cancer cells to fulvestrant via ERK/HIF‑1 pathway inhibition *in vivo*

**DOI:** 10.3892/mmr.2024.13357

**Published:** 2024-10-09

**Authors:** Xiaoqing Jia, Jingyi Cheng, Zhenzhou Shen, Zhimin Shao, Guangyu Liu

Mol Med Rep 17: 5470–5476, 2018; DOI: 10.3892/mmr.2018.8514

Following the publication of the above article, the authors drew to the Editor's attention that they had inadvertently used the same immunohistochemical image to show the experiments depicting the zoledronic acid-treated MCF-7/HIF-1α xenograft (the ‘ZOL/MCF-7/hif’ panel) and the fulvestrant-treated MCF-7/vector xenograft (the ‘FUL/MCF-7/cdh’ panel) in Fig. 3A on p. 5474. Subsequently, upon performing an independent review of the data in this paper, the Editorial Office pointed out to the authors that the same colony-formation assay image had been included in Fig. 1C to show the ‘MCF-7/cdh-ZOL’ and ‘MCF-7/cdh-FUL’ experiments.

The authors re-examined their original data, and realized that inadvertent errors were made during the compilation of this pair of figures. The corrected versions of Figs. 1 and 3 are shown on the next two pages, now featuring the correct data for the ‘MCF-7/cdh-ZOL’ experiment in Fig. 1C and the ‘ZOL/MCF-7/hif’ experiment in Fig. 3A. All the authors agree with the publication of this corrigendum, and are grateful to the Editor of *Molecular Medicine Reports* for granting them the opportunity to publish this. Furthermore, they regret that these errors were introduced into the paper, even though they did not substantially alter any of the major conclusions reported in the paper, and apologize to the readership for any inconvenience caused.

## Figures and Tables

**Figure 1.**
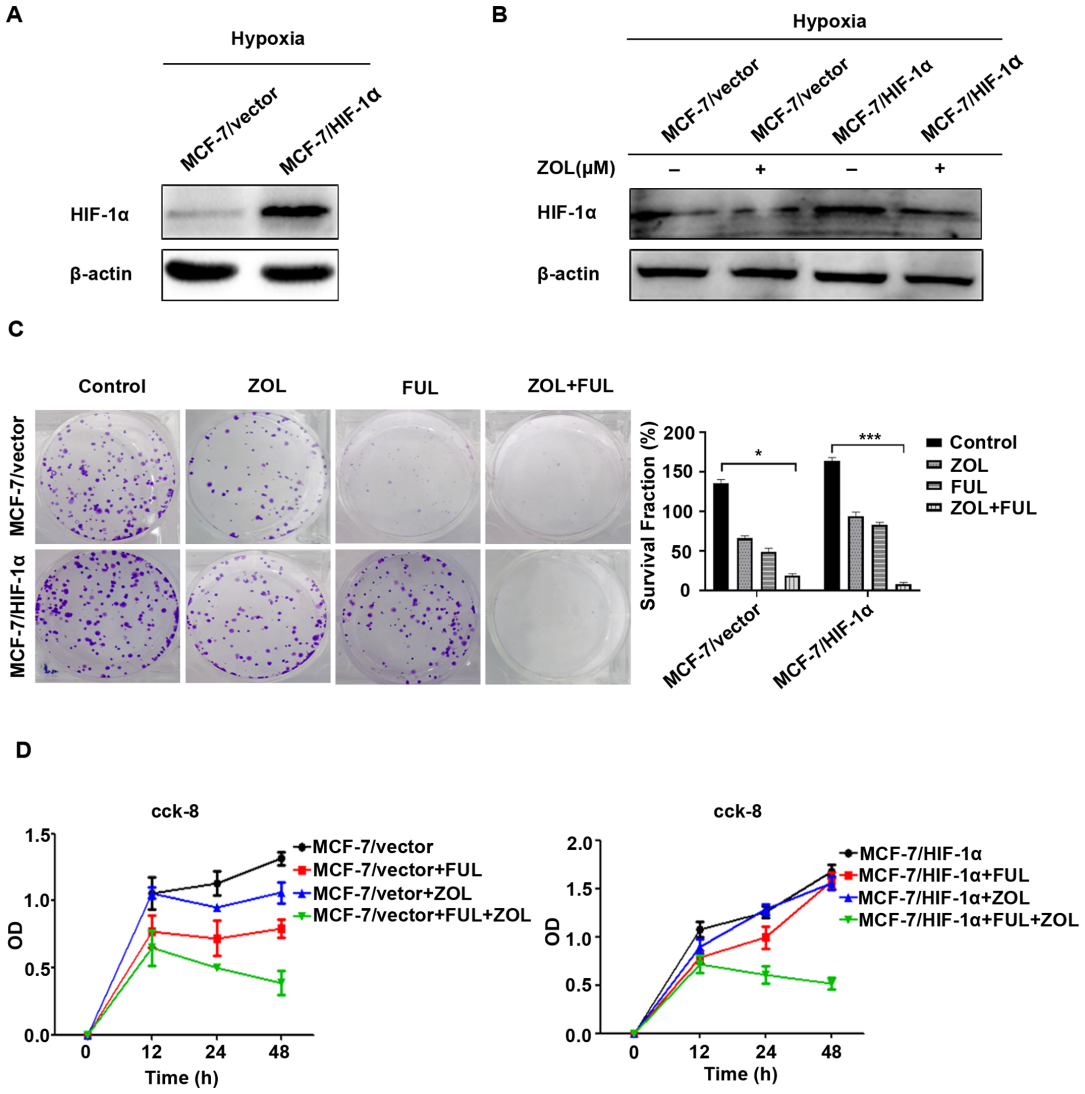
ZOL exerts antitumor activity in HIF-1α-overexpressing breast cancer cells and synergizes with fulvestrant *in vitro*. (A) Western blot analysis of the control MCF-7/vector and MCF-7/HIF-1α cells, demonstrating that HIF-1α overexpression was successfully established. (B) MCF-7/HIF-1α and MCF-7/vector cells were pre-treated with 200 µmol CoCl2 for 6 h followed by treated with CoCl2 and 100 µmol ZOL for 18 h and western blot analysis was used to detect HIF-1α expression. (C) Growth of MCF-7/HIF-1α and MCF-7/vector cells treated with 100 µmol ZOL and/or 0.1 nmol/l fulvestrant for two weeks, as determined by colony formation assay. Control is untreated cells. (D) Viability of MCF-7/HIF-1α and MCF-7/vector cells treated with 100 µmol ZOL and/or 0.1 nmol/l fulvestrant for 0, 12, 24, 48 h, as determined by Cell Counting Kit-8 assay. *P<0.05 and ***P<0.001 vs. untreated control. ZOL, zoledronic acid; HIF, hypoxia-inducible factor; FUL, fulvestrant; OD, optical density.

**Figure 3.**
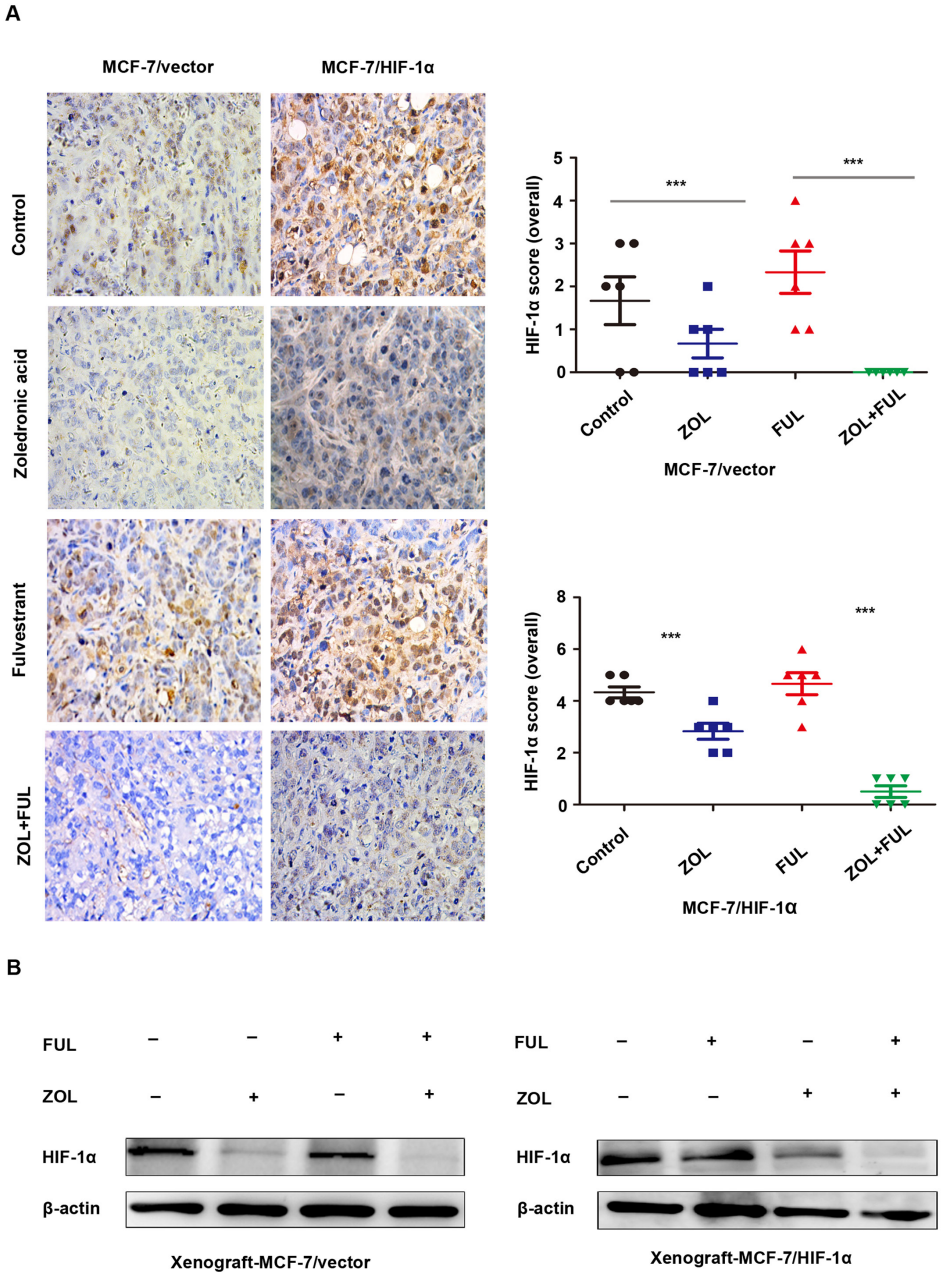
ZOL inhibits HIF-1α expression *in vivo*. (A) Immunohistochemical analysis of HIF-1α expression in xenograft tumor tissues from the four experimental groups. Representative images (magnification, ×200) and quantification. (B) Representative images from western blot analysis of HIF-1α expression in xenograft tumor tissues from the four experimental groups. ***P<0.001 vs. untreated control. ZOL, zoledronic acid; HIF, hypoxia-inducible factor; FUL, fulvestrant.

